# Parents’ and carers’ impression of “quality” within a Paediatric Emergency Department

**DOI:** 10.1186/s12887-021-02752-7

**Published:** 2021-07-13

**Authors:** Brendan Lacey, Adam West, Simon Craig

**Affiliations:** 1 Paediatric Emergency Department, Emergency Service, Monash Health, Monash Medical Centre, 246 Clayton Road, Clayton, Victoria 3168 Australia; 2 Department of Medicine, School of Clinical Sciences, Monash University, Clayton, Victoria Australia; 3 Monash Children’s Hospital, Locked Bag 29, Clayton South, Victoria Australia

**Keywords:** Quality assurance, Paediatrics, Emergency department, Feedback, Patient satisfaction

## Abstract

**Background:**

Quality improvement systems are needed to overcome the ‘Quality Gap’ – difference between evidence-based guidelines and the care delivered. While there are a large array of potential quality assurance measures exists in the Paediatric Emergency Department, parent’s/carer’s perception of these is unknown. This study aimed to identify what ‘quality of care’ means to parents/carers of Paediatric Emergency Department (PED) patients, further determine which aspects of these are most important to them. Also, to identify which of the existing PED quality measures are most important to parents/carers, and their preferred method of providing feedback.

**Methods:**

A Modified Rand-Delphi study was performed with parents/carers as the expert group and consensus was obtained from them via three web-based surveys. All parents/carers of children attending a tertiary paediatric hospital during six-week in winter were eligible– no exclusions. Quality measures scoring at least 7 on a 9-point Likert scale during the final survey were considered “very important”, while those scoring at least an 8 were considered “extremely important”.

**Results:**

One hundred four parents/carers responded from a total of 1095 participants. Parents/carers generated 527 free text entries, to the initial survey on what ‘quality of care’ means. These were mapped to 48 quality measure which they ranked on subsequent surveys. Eighteen quality measures were considered very important by at least 90% of respondents. Of these, six were considered extremely important by at least 70% of respondents: ‘Thorough medical assessment’ (84%); ‘A triage system’ (84%); ‘Experienced and knowledgeable staff that are skilled in paediatrics’ (77%); ‘Resources and equipment available to provide care’ (72%); and ‘Clear follow up plans and reviews that are communicated and scheduled’ (72%). Parents/carers considered existing quality measures as important with ‘timely treatment of a critical condition’ as the most important. Most participants preferred to provide anonymous feedback (*N* = 69, 66%), online (*N* = 77, 72%) after discharge (*N* = 82, 70%).

**Conclusion:**

We have elicited what ‘quality of care’ means to parents/carers, and which aspects are most important to them. Parents/carers consider commonly used PED quality measure as very important. However, they are less important than outcomes generated by themselves. Further parents/carers in this study preferred to provide feedback that was anonymous and electronically distributed after they leave the ED.

**Supplementary Information:**

The online version contains supplementary material available at 10.1186/s12887-021-02752-7.

## Background

Quality in healthcare is the degree by which a health service increases the likelihood of the desired health outcome for an individual and population. The “Quality Gap” is the difference between evidence-based guidelines and the care delivered [1], and has been the impetus for regulatory and professional bodies around the world to mandate quality assurance or quality improvement processes [2, 3]. Currently in Australia the Paediatric Hospital Medicine Dashboard model (a derivative of the Institutes of Medicine (IOM)‘s quality domains [4]) is the most commonly used framework to benchmark paediatric hospital performance [5].

To date, there has been a large body of research in Paediatric Emergency Department (PED) quality assurance measures resulting in an array of general and disease specific quality assurance parameters (see Supporting Information Table A & B) [6–11]. General quality assurance parameters address issues such as workload, waiting times, safety, patient feedback and effectiveness. Disease-specific quality measures refer to activities relevant to a particular disease, such as time to anticonvulsant medication in a child with seizures, or appropriate use of bronchodilators for asthma. Parameters relevant to high acuity conditions are ranked highly by PED clinical leaders [9], while health system function and flow measures are particularly relevant to hospital administrators. However, it is unknown whether these measures reflect parents/carers’ priorities.

Research on paediatric inpatient admissions show that parents/carers consider caring staff, effective communication, safety and the physical environment as key aspects of satisfaction [12]. Similarly, their satisfaction with a PED relates primarily to medical team communication, waiting times, pain management, and perceived quality of medical care [13]. Currently there are a range of paediatric Patient Reported Outcomes Measurements (PROMs), such as ‘General Health’, ‘Physical Health’, ‘Mental Health’, ‘Feeling sad’, ‘Fun with Friends’, ‘Parents listen’ and ‘Quality of Life’ to measure clinical outcomes [14]. These are very generic and do not reflect the care that is delivered in the PED.

The aim of this study was to determine the perspective of parents/carers on the quality of care their child received at a busy emergency department. Our specific objectives were to:

- Identify what ‘quality of care’ means to parents/carers of PED patients, and what aspects of this quality of care are most important to them.
- Determine how important parents/carers consider current PED quality assurance metrics to be; and
- Determine parents’/carers’ preferred method for providing feedback about their PED visit.

### Article summary - brief points

- What is already known on this topic:
  - ◦ Quality improvement systems are needed to overcome the ‘Quality Gap’ – difference between evidence-based guidelines and the care delivered.
  - ◦ A large array of potential quality assurance measures exists in the Paediatric Emergency Department.
  - ◦ Email/website-based surveys are a valid method of collecting patient feedback in the healthcare setting.
- What this paper adds:
  - ◦ Parents/carers consider thorough and optimum care, skilled paediatric staff with resources to provide care, a triage system, and clear follow-up plans, as most important.
  - ◦ Parents/carers of PED patients prefer to provide anonymous feedback online after discharge from hospital.
  - ◦ Parents/carers rated commonly used clinical quality assurance parameters as very important, with the timely treatment of high acuity conditions considered most important.

### Article summary - strengths and limitations of this study

- This study targeted and asked parents/carers as the “Expert Group” to garner what was important to them with regards to the care their child received in a busy paediatric Accident and Emergency department.
- The modified Rand-Delphi process allowed us to gain a consensus agreement from parents /carers on the difficult to define and context dependent issue of quality of care in a busy paediatric Accident and Emergency department.
- This study also assessed and got consensus on what parents/carers thought of current paediatric Accident and Emergency department quality assurance metrics.
- A limitation of this study was its low response rate; however, the response rate was equivalent to studies with similar methodologies.
- Other limitations of the study are its inherent bias of excluding non-English speaking parents/carers, those of limited technical literacy, and requiring participants to have access to the internet.

## Methods

**Fig. 1:**
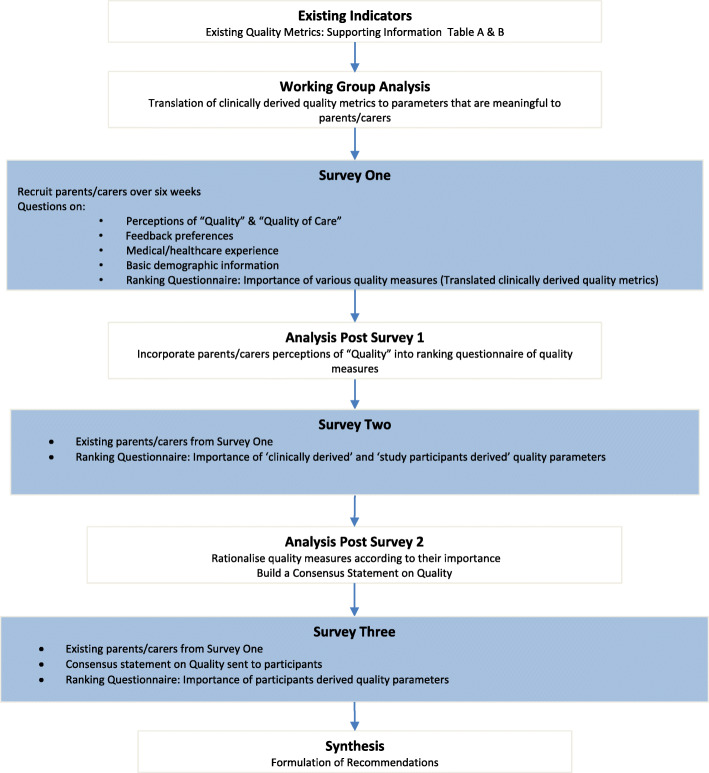
Study Design

Parents/carers of PED patients were invited to participate in a modified Rand-Delphi process [15]. This involved a number of iterative phases, until a consensus was reached (Fig. 1). Three sequential online surveys were distributed to parents/carers recruited from the ED. After each survey the outcomes were analysed and used to inform the next phase of the study. Further, a consensus statement of results was also sent to the parents/carers prior to the third survey. This allowed participants to reflect upon the responses of others before finalising their reply [15].

There were two free text questions in Survey One in which the parents and carers were asked to provide their impression of quality:

- ‘In the Emergency Department we try and give the best care to the children who need our service. What does ‘best care’ mean to you?’
- ‘How could we make the care we give to you and your child even better?’

An initial working group was formed to translate the clinician-derived quality assurance parameters (See Supporting Information Table A & B) into parent/carer-friendly terms (removing medical jargon and repetition). This group consisted of the project team, PED clinical leadership, a representative from the healthcare organisation’s ‘Patient & Family Experience Office’ and a consumer representative. This team was formed by the PED’s research coordinator liaising with the healthcare organisation ‘s research support services. Team members were selected as per the organisations ethics and research support polices and their availability. All of these clinician-derived assurance parameters were mapped to simplified parameters and then then ranked by survey participants via a 9-point Likert scoring system (1 - ‘Not at all important’ to 9 -‘Extremely important’). The aggregated data for each parameter was then classified by the following criteria [15]:

- A score 8–9 by > 70% of participants: Extremely important
- A score 7–9 by > 70% of participants: Very important
- A score 4–6 by > 70% of participants: Important but not critical
- A score 1–3 by > 70% of participants: Limited importance

The Modified Rand-Delphi process builds consensus on difficult to define topics. In this case, the aspect of quality considered most important by parents/carers. Thereby, only ‘Very Important’ and ‘Extremely important’ parameters were carried forward at each stage. No parameters in this study were classified as ‘Limited importance’.

This study was performed at a single metropolitan tertiary PED, over a six-week period from August to September 2018 (southern hemisphere winter). The participants were the parents/carers of PED patients and all were invited to in the study with no exclusions. Parents/carers were given printed information statements about the study at patient registration. This was followed with an oral discussion with clinical staff those interested in participating provided their email and written consent to the clerical or clinical staff. Parents/carers completed an electronic questionnaire within 1–5 days of presentation, for each of the three survey phases sent via email with the SurveyMonkey online tool. If the survey was not completed participants received a reminder on the 5th and 10th day after sending the survey. Each survey remained open for 3 weeks and participants who completed the earlier phase were used for the next phase. Survey Two occurred 4 weeks after the initial survey, and Survey Three occurred 2 weeks after the completed of Survey two.

Qualitative analysis of participants’ open-ended questions utilised thematic content analysis with an open coding framework [16]. One author (BL) analysed and read all the ‘Free text’ responses, identified themes and patterns in the material. Categories were created to capture all aspects of the material (similar content and responses were grouped together under their parent category). The responses were then repeatedly reread and analysed until all responses were categorised and a frequency given to each category - detailed breakdown of this analysis can be seen in Supporting Information Table C. A single study member performed the analysed for consistency and all material was then reviewed by the project team.

This study used basic descriptive statistics such as number and percentage for categorical data. The Chi square test was to assess any difference between the study participants and the PED population in relation to age distribution and sex.

Ethics approval for this study was obtained hospital network’s Human Research Ethics Committee. This research received no specific grant from any funding agency in the public, commercial or not-for-profit sectors.

### Patient and public involvement

The project working group consisted of a representative from the healthcare organisation’s ‘Patient & Family Experience Office’ and a consumer representative. These members helped design the ranking questions and assurance parameters into carer friendly/easy to understand terms for the modified Rand-Delphi process. Patients were NOT involved in the recruitment or conduction of the study but were naturally participants. Results were disseminated to ALL study participants during the consensus building stage as well in the final synthesis and formulation of recommendations. All communications were via email.

## Results

**Fig. 2:**
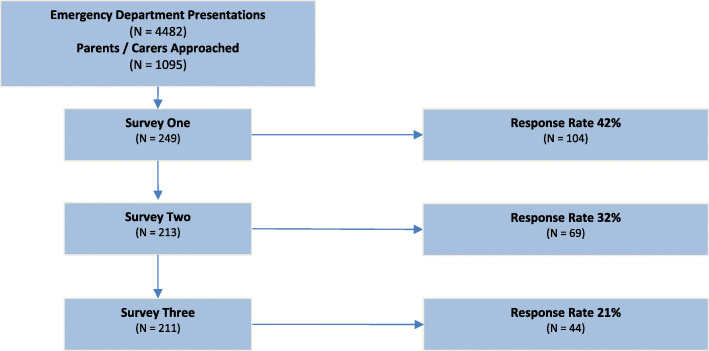
Response rate for each survey

One thousand ninety-five study information leaflets were distributed during the six-week study period. 249 (23%) parents / carers provided contact details. We received 104 responses to Survey One, 69 to Survey Two and 44 to Survey Three, giving response rates of 42, 32 and 21% respectively (Fig. 2). The age and gender distribution of children amongst survey one respondents were similar to the overall population of children attending the ED during the study period (Table 1). Most respondents were mothers (83%), most had attended the ED at least once before (74%), and most children did not have a chronic medical condition (80%) (Table 1).

**Table 1:** Demographic characteristics of study participants and ED population during the study

	*Survey Respondents*		*All ED attendances*		*Chi Square Results (p)*
	*No.*	*Percentage (%)*	*No.*	*Percentage (%)*	
*Age of Child*					*0.999*
*< 12 months old*	*7*	*6.8%*	*731*	*16%*	
*1–5 years*	*48*	*46%*	*1669*	*37%*	
*6–12 years*	*30*	*29%*	*1360*	*30%*	
*13–18 years*	*17*	*16%*	*641*	*14%*	
*18+ years*	*1*	*1.0%*	*81*	*1.8%*	
*Sex of Child*					
*Male*	*48*	*46%*	*2495*	*56%*	*0.855*
*Female*	*55*	*54%*	*1984*	*44%*	
*Who brought the patient/child to the emergency department?*					
*Mother*	*87*	*82%*			
*Father*	*14*	*14%*			
*Sibling*	*1*	*1.0%*			
*Grandparent*	*0*	*0.0%*			
*Other (Not stated)*	*2*	*3.9%*			
*Does the child have a Chronic Medical condition?*					
*Yes*	*21*	*20%*			
*The number of times the child has presented to ED before*					
*0–1*	*27*	*26%*			
*2–3*	*48*	*47%*			
*4–5*	*20*	*20%*			
*6 +*	*7*	*7%*			

The open-ended questions in Survey One yielded 527 free text entries, which were translated to 48 quality measure categories (some responses were relevant to multiple categories). Any category that achieved > 0.2% of responses (≥2 respondents) was then mapped to a ranking question in Survey Two as per the working group analysis (generating 32 survey questions). Some parameters were mapped to multiple questions and in these situations the question providing the highest Likert score was used for analysis. The results of the consensus phase were that nearly all parent/carer-defined quality parameters were considered “Very important”, with the notable exception of ‘Shorter wait times’ and ‘Minimal bureaucracy, repetition and administration’ (Table 2). Six parameters were considered ‘Extremely important’.

**Table 2:** Consensus on the top 20 most important aspects of “Quality” to parents/carers – highlighted judged the most important

Commentary on best care/quality (*n* = number of responses)	Final Consensus Survey	
	% Likert Score Very Important (Score 7–9)	% Likert Score Extremely Important (Score 8–9)
Thorough and optimum medical assessment (*n* = 43)	98% (*n* = 42)	84% (*n* = 36)
Treating the sicker kids first (*n* = 43)	100% (*n* = 43)	79% (*n* = 34)
Experienced and knowledgeable staff (*n* = 44)	100% (*n* = 44)	77% (*n* = 33)
Specialised nurses and doctors for children (*n* = 44)	100% (*n* = 44)	77% (*n* = 33)
Resources and equipment availability (*n* = 43)	88% (*n* = 38)	72% (*n* = 31)
Clear follow up plans and reviews that are communicated and scheduled (Including ED, GP and Outpatients) (*n* = 43)	95% (*n* = 41)	72% (n-31)
Retain experienced and skilled paediatric staff (*n* = 43)	98% (*n* = 42)	68% (*n* = 29)
Staff should act professionally and support each other (*n* = 44)	91% (*n* = 40)	66% (*n* = 29)
Triage assessment should be performed in a timely manner (*n* = 43)	95% (*n* = 41)	63% (*n* = 27)
Triage to provide initial care such as pain relief (*n* = 43)	95% (*n* = 41)	63% (*n* = 27)
Review and check children while they are waiting (*n* = 43)	93% (*n* = 41)	61% (*n* = 27)
Medical Treatment followed (*n* = 44)	89% (*n* = 39)	61% (*n* = 27)
Timely access to sub specialists if needed (*n* = 43)	93% (*n* = 40)	61% (*n* = 26)
Timely management of all children (examination, investigations and treatment) (*n* = 44)	95% (*n* = 42)	57% (*n* = 25)
Staff listen to, understand parents and carers and have exceptional communications skills (*n* = 44)	93% (*n* = 41)	55% (*n* = 24)
Feedback and updates to the carer/parents on the progress of care (investigations and treatment). (*n* = 43)	98% (*n* = 42)	54% (*n* = 23)
Medical terms and results should be explained in simple language (*n* = 43)	93% (*n* = 40)	54% (*n* = 23)
Engage parents / carer in the care and treatment of their child (*n* = 44)	91% (*n* = 40)	52% (*n* = 23)
Kind, caring and empathic staff who are friendly, courteous and compassionate (*n* = 43)	84% (*n* = 36)	51% (*n* = 22)
Timely review of patient to assess progress (*n* = 43)	84% (*n* = 38)	49% (*n* = 21)

Participants ranked the clinically derived parameters in Survey One and Survey Two and considered all clinically derived parameters as ‘Very important’. One parameter - related to the timely treatment of status epilepticus - was considered ‘Extremely important’ (Table 3).

**Table 3:** Consensus phase Clinically Derived Parameters ranking (Extremely Important parameters highlighted)

Quality Assurance Measure for Specific Conditions	Final Consensus Survey	
	% Likert Score Very Important (Score 7–9)	% Likert Score Extremely Important (Score 8–9)
**Patient Centred**		
•Customer feedback “Excellent/Very Good /Good” (*n* = 68)	**79%** (*n* = 54)	**35%** (*n* = 24)
•Discharge summary completed within 48 h (*n* = 68)	**85%** (*n* = 58)	**50%** (*n* = 34)
•Median time to complete discharge summary (*n* = 68)	**85%** (*n* = 58)	**50%** (*n* = 34)
•Complaints (*n* = 68)	**79%** (*n* = 54)	**35%** (*n* = 24)
**Safety**		
•Child protection screening (*n* = 68)	**85%** (*n* = 58)	**50%** (*n* = 34)
Asthma		
•Time to reliever treatment (β_2_ agonist/Ipratropium**)** (*n* = 68)	**91%** (*n* = 62)	**56%** (*n* = 38)
•Time to steroids (> 5 yrs. & Moderate/Severe/Critical) (*n* = 68)	**91%** (*n* = 62)	**56%** (*n* = 38)
•Discharged with action plan & education (*n* = 68)	**85%** (*n* = 58)	**50%** (*n* = 34)
•Discharged with steroids (*n* = 68)	**85%** (*n* = 58)	**50%** (*n* = 34)
•Discharged with preventer (*n* = 68)	**85%** (*n* = 58)	**50%** (*n* = 34)
•Discharged with follow-up (*n* = 68)	**85%** (*n* = 58)	**50%** (*n* = 34)
Neonatal Sepsis/Meningitis		
•Time to antibiotics (*n* = 68)	**91%** (*n* = 62)	**56%** (*n* = 38)
•% of patient requiring bolus given within 1 h (*n* = 68)	**91%** (*n* = 62)	**56%** (*n* = 38)
•% patient refractory shock requiring inotrope (*n* = 68)	**91%** (*n* = 62)	**56%** (*n* = 38)
Status Epilepticus		
•Time from arrival & % patient received benzodiazepine in ED (*n* = 68)	**87%** (*n* = 59)	**60%** (*n* = 41)
•Time from arrival to second line anti-epileptics (*n* = 68)	**87%** (*n* = 59)	**60%** (*n* = 41)
•% Patient & Time to initial BSL (*n* = 67)	**90%** (*n* = 60)	**50%** (*n* = 34)
•% patient failure to achieve seizure control within 30mins (*n* = 68)	**90%** (*n* = 10)	**72%** (*n* = 49)
Severe Head Injury		
•Median time to imaging from request (*n* = 68)	**91%** (*n* = 62)	**56%** (*n* = 38)
•Median time to neurosurgeon response from request (*n* = 68)	**91%** (*n* = 62)	**56%** (*n* = 38)
•Median time to definitive airway management (*n* = 68)	**91%** (*n* = 62)	**56%** (*n* = 38)

The vast majority of respondents (75%) preferred an email/website-based feedback mechanism and for it to take place post discharge (70%) (Table 4). The majority (66%) of respondents preferred to be anonymous (Table 2).

**Table 4:** Parent/carer’s feedback preferences

Feedback preferences		
Mechanism	Number	Percentage
Phone Call	9	8.7%
Paper survey	5	4.9%
Email / Website survey	77	75%
Smart phone app	7	8.7%
Feedback kiosk at the hospital entrance	3	2.9%
Other (please specify)	0	0%
Timing (Multiple responses allowed *n* = 118)		Percentage
As inpatient	18	15%
At discharge	17	14%
Post Discharge (within 2 days of hospital discharge)	40	34%
Post Discharge (within a week of hospital discharge)	42	36%
Other	1	0.8%
		Percentage
Preferred Feedback to be Anonymous	69	66%

## Discussion

Parents and carers attending the ED make judgements about the quality of care they and their child receive. Our Modified Rand-Delphi study has demonstrated that parents/carers consider a large number of quality measures very important. A smaller number of parameters were considered ‘Extremely important’; these addressed broad aspects of clinical care: prioritisation of high-acuity patients, excellent care delivered by well-trained and experienced staff working in an appropriately resourced setting, and adequate follow-up arrangements on discharge.

Clear communication with follow-up plans is a consistent finding in adult [17] and paediatric studies [18], and was found to be one of the most important aspects of care in our cohort. Improved communication and education in the PED, as well as increased clinic access (i.e. General Practitioner), decreases low acuity PED presentations and improves healthcare efficiency [19]. Other extremely important factors highlighted in our study were, thorough and optimal medical care in combination with experienced and skilled staff. These findings are consistent with adult ED research which emphasizes trust in clinical competence [17], similarly paediatric studies prioritise the perceived quality of medical care [13]. The importance of resources and equipment available to provide care is consistent with adult and paediatric studies that highlight safety and the physical environment as key aspects of care [12, 17]. The final quality factor that parents/carers considered the most important was treating sicker children first. This can be interpreted as consensus between parents/carers and clinicians who rate time-critical treatments as most important [6, 9].

Parents/carers consider current PED quality assurance metrics [6] to be ‘Very important’, with the most highly ranked measure being one related to time critical intervention in a high-acuity condition. Although time-based parameters such as shorter waiting time, timely treatment and providing updates about waiting featured heavily in open-ended responses, they were not considered extremely important. There by many quality metrics used by the PED while valuable do not necessarily reflect parent/carer priorities.

Interestingly, shorter waiting times were not even considered ‘Very important’. While this is at odds with some studies [13], it is consistent with adult Emergency Departments - where shorter waiting time did not feature in the top 20 most important aspects of quality care [17]. It is also consistent with previous findings which showed that the length of the wait times did not negatively influence overall parents’/carers’ satisfaction with PED services in Australia [20].

Translating these parent/carer-derived factors into quality assurance parameters require feedback from parent/carers. A key component will be patient-reported experience measures (PREMs) questionnaires which capture these most important domains (‘Extremely important’ in Table 2). While PREM questionnaires may not have the ideal validity, reliability and responsiveness [21], they are sufficient for quality improvement [22]. Thus, a PED quality assurance system should incorporate PREMs questionnaires covering these domains. Namely a short post discharge anonymous patient questionnaire as suggested in Table 5. This could utilize a combination of Likert rating scale and binary (yes/no) questions. Achieving PREM in a busy PED is difficult without a patient portal or standardised feedback questionnaires. Web-based applications (such as KILK, http://www.hetklikt.nu [23]), and customer feedback kiosks [24] have provided an interface to capture this information. The Victorian healthcare Experience Survey captures a detailed picture of patient experiences and satisfaction with the services received [25]. However the survey’s length and response rate (< 15% for Paediatrics) limits its day to day usefulness [26]. A logical progression of these technologies is a smart device application which implements PREM. However there is a substantial technology and systems infrastructure gap at the hospital level that inhibits its implementation [27].

**Table 5:** Possible Patient reported experience measure (PREM) to capture the most important quality domains as found in this study

Parent/Carer Quality Assurance Domain	PREM	
	Likert Rating Scale questions (Score 0–9)	
	Strongly Disagree	Strongly Agree
Thorough and optimum medical assessment	“The medical care my child received was thorough and to a high standard”	
Treating the sicker kids first	“Sicker children and those needing urgent care receive it first”	
Experienced and knowledgeable staff & Specialised nurses and doctors for children	“The staff were experienced, knowledgeable, and had the skill required to care for children”	
	**Binary Questions**	
Resources and equipment availability	“Was there enough resources and equipment available in the Emergency Department to provide care?” Yes □ No □	
Clear follow up plans and reviews that are communicated and scheduled (Including ED, GP and Outpatients)	“Were clear follow-up plans made and review scheduled if needed?” Yes □ No □	

Participants of this study preferred email/website-based feedback – which is perhaps a non-surprising response from an email/website-based survey. While a self-selecting sample bias cannot be excluded, it was clearly the most popular with 75% of respondents; all others including phone call, smart device app and feedback kiosk were under 10%. There is growing evidence that email/website-based surveys are a valid method of collecting patient feedback [28]. Our study showed a clear majority of respondents (84%) prefer to provide feedback at discharge or after – mostly within 2 days to a week of leaving hospital. This does not lend itself to personalised feedback to individual clinical staff - as the context and granularity of those individual patients are lost in high volume and short duration encounters [29]. Also, of all respondents, the majority (66%) preferred to be anonymous. This timing and anonymity paradox is well described [30]. Nonetheless our findings that parents/carers wish for feedback to be anonymous, email-based and within 2 to 7 days post discharge, does lend itself to a whole-of-department quality improvement system.

### Limitations

A significant limitation of our study is a low response rate, which has been found in similar ED studies [31, 32]. Possible reasons for the low recruitment rate include lack of dedicated research staff (clerical staff were asked to distribute the study information in addition to their usual duties) and low priority of the study in a busy department. Improvements in communication and study design (dedicated research staff) would aid recruitment. Poor recruitment increased the risk of participation/non-responder bias. However, this can be considered less likely given the age and sex profile of the children of the recruited participants matched the profile of ED presentations. Also given the anonymous nature of the study, data is not known on the triage category, compliant/diagnosis, wait times, or disposition of the child and how these may influence the parents/carers response. Other limitations of the study are its inherent bias of excluding non-English speaking parents/carers (40% of the healthcare organisation’s population are born overseas and 38% speak a language other than English at home [33]), those of limited technical literacy and the socio-economic status needed to access the internet. Furthermore, it is known that internet-based surveys have a lower response rate which can deteriorate with an increasing burden of electronic correspondence [34]. Nonetheless the low cost, rapid response, need for repeated surveys, ease of collation of results and feedback as well as data integrity, were thought to out-weigh these disadvantages for this study.

## Conclusion

Thorough and optimum medical care, skilled paediatric staff with resources to provide care, a triage system, and clear follow-up plans were the most important aspect of quality of care for the parents/carers of children attending a paediatric emergency department.

Parents/carers rated commonly used clinical quality assurance parameters as very important, with the timely treatment of high acuity conditions most important. Participants also preferred feedback to be anonymous, occur post discharge and to be email/web-based.

The addition of more patient-centred domain into the paediatric ED quality assurance system such as those suggested in this study and that are anonymous and electronically distributed after parents/carers leave the ED, are likely to enhance the healthcare organisation quality assurance systems.

## Supplementary Information

**Additional file 1.**

## Data Availability

All data generated or analysed during this study are included in this published article (and its supplementary information files).
