# Supplementary material for: Parents’ and carers’ impression of “quality” within a Paediatric Emergency Department
Source: BMC Pediatr. 2021 Jul 13;21:313. doi: 10.1186/s12887-021-02752-7 (PMC8276454; doi:10.1186/s12887-021-02752-7)
Supplement: Supplementary file 1 — Additional file 1. [file 12887_2021_2752_MOESM1_ESM.pdf]

Parents' and Carers' Impression of "Quality" within a Paediatric Emergency Department

Supporting Information

Table A General ED Quality Assurance parameters

Workload

Workload General

- Number of Presentations
- Number of patients who Did not wait (DNW) for treatment
- Number of Admissions

Workload Acuity

- Number & percentage Triage Category 1 / 2 / 3 / 4 / 5
- Median time in resuscitation Area Triage Category 1&2

Workload Timely Disposition

- Number & percentage of presentation seen in <4hrs
- Median time to disposition
- Triage Category 1 / 2 / 3 / 4 / 5
  - Presentations seen in <4hrs
  - Median time to be seen
  - Median time to disposition

Workload Capacity

- Number of staff (nursing / physician/ allied health)
- Number of beds open (ED & ED Observation)
- Median length of stay (ED & ED Observation)

Patient Centred

- Percentage of patients who leave without been seen
- Customer feedback "Excellent/Very Good /Good"
- Discharge summary completed within 48hrs

• Median time to complete Discharge summary<br>• Time to be seen<br>• Time to initial Treatment, including analgesia<br>• Complaints
**Safety**<br>• Number of medication errors<br>• Handover related errors<br>• Treatment or investigation errors<br>• Child protection screening
**Effectiveness & Efficiency**<br>• Time to analgesia<br>• Representation to ED in 48hrs (excluding scheduled reviews)<br>• Representation to ED in 48hrs requiring admission (excluding scheduled reviews)

Table B Disease Specific Quality Assurance measures

Quality Assurance Measure – Disease specific

Asthma

- Time to reliever treatment (β_2 agonist/Ipratropium**)**
- Time to steroids (>5yrs & Moderate/Severe/Critical)
- % Patients received steroids
- % Patients received respiratory assessments/grading
- % Patients received second line agent
- % Patients received Ipratropium
- % Patients received Aminophylline / Magnesium Sulphate
- Discharged with Action plan & Education
- Discharged with steroids
- Discharged with Preventer
- Discharged with follow-up
- Steroids given in <5yrs and mild asthma
- Chest x-rays in acute asthma

Bronchiolitis

- Total number of bronchiolitis presentations
- Dispositions admission/home/scheduled review/representations <48hrs
- Number of chest x-rays

Neonatal Sepsis / Meningitis

- Time to antibiotic administration
- Time to investigations
- Time to disposition

Severe Sepsis / Shock

- Time to IV/IO access
- Time to antibiotics administration
- Median time to fluid bolus
- % of patients requiring bolus given within 1hrs
- % of patients with refractory shock requiring inotrope

• % of patients Initial blood glucose in 1 hr
Oncological Febrile Neutropenia<br>• Time to investigations (FBE)<br>• Time to Antibiotics<br>• Time to disposition
Severe Head Injury<br>• % of Patients without spinal precaution<br>• % Patients with untreated hypotension<br>• % Patients receiving neuro observations including BP<br>• Median time to imaging from request<br>• Median time to neurosurgeon response from request<br>• % Needing definitive airway management<br>• Median time to definitive airway management
Status Epilepticus<br>• Time from arrival & % of patients who received benzodiazepine<br>• Time from arrival to second line anti-epileptics<br>• % Patient & Time to check initial blood glucose<br>• % patient failure to achieve seizure control within 30mins
Diabetes Ketoacidosis (DKA)<br>• % Patient received IV Normal saline<br>• % Patient received appropriate insulin dose & route<br>• % patient received potassium replacement<br>• % patient received IV insulin<br>• % patient received bicarbonate
Anaphylaxis<br>• % patients received adrenaline<br>• % patient received adrenaline by appropriate route
Fractures Triage category 1 / 2 / 3<br>Time to Analgesia in ED (include analgesia via Ambulance service)

Table C Analysis of participant's impression of "Quality"

Commentary on best care/quality	No of responses	Percentage of Total (%)	Survey Two		Survey Three	
			% Likert Score Very Important (Score 7 - 9)	% Likert Score Extremely Important (Score 8 - 9)	% Likert Score Very Important (Score 7 - 9)	% Likert Score Extremely Important (Score 8 - 9)
**Kind, caring and empathic staff who are friendly, courteous and compassionate**	69	13~~.1~~%	94~~.2~~ %	61~~0.9~~%	89~~.3~~%	51~~.2~~%
• **Caring & loving**	18	3.4%				
• **Friendly**	17	3.2%				
• **Kind**	10	1.9%				
• **Understanding**	6	1.1%				
• **Patient**	5	1.0%				
• **Compassionate**	5	1.0%				
• **Empathy**	4	0.8%				
• **Supportive**	4	0.8%				
**Timely management of children (assessment, diagnosis and treatment)**	60	11~~.4~~%	95~~.00~~%	61~~0.90~~%	96~~5.50~~%	57~~6.80~~%
**Thorough and optimum medical assessment**	56	11~~0.6~~%	97~~.1~~%	80~~79.7~~%	98~~7.7~~%	84~~3.7~~%
**Staff should listen to and understand parents and have exceptional communication skills**	45	8.6%	94~~.2~~%	51~~0.7~~%	93~~.2~~%	55~~4.6~~%
**Staff should provide feedback & updates to carers/parents on the progress of care (investigations and treatments)**	44	8.4%	97~~.1~~%	63~~2.9~~%	98~~7.7~~%	54~~3.5~~%
**Spacious / uncrowded environment waiting area and ED**	42	8.0%	87~~.0~~%	45~~4.9~~%	7~~6.~~7%	35~~4.9~~%
**Explaining medical conditions, results and plans should be done in simple terms**	39	7.4%	94~~.2~~%	54~~3.6~~%	93~~.0~~%	53~~.5~~%
**Experienced and knowledgeable staff**	31	5.9%	97~~.1~~%	76~~5.5~~%	100%	77~~.3~~%
**Shorter wait times**	29	5.5%	74~~3.9~~%	33~~.3~~%	68~~.2~~%	32~~1.8~~%
**Tailored child friendly environment – waiting area and ED**	25	4.8%	87~~.0~~%	49~~.3~~%	81~~.4~~%	42~~1.9~~%
**Clear follow up plans and reviews that are communicated and scheduled (Including ED, GP and Outpatients)**	23	4.4%	99~~8.5~~%	60~~.3~~%	95~~.4~~%	72~~.1~~%
**Triage assessment should be performed in a timely manner**	22	4.2%	100%	65~~.2~~%	95~~.4~~%	63~~2.8~~%
**Resources and equipment availability**	20	3.8%	94~~.2~~%	73~~2.5~~%	88~~.4~~%	72~~.1~~%
**All staff behaving in a calm and professional manner that communicates to and supports each other**	20	3.8%	97~~.1~~%	78~~.3~~%	91~~0.9~~%	66~~5.9~~%
**More medical & nursing staff in the Emergency department to treat patients**	18	3.4%	78~~.3~~%	55~~.1~~%	86~~.4~~%	52~~.3~~%
**Timely review of patient to assess progress**	15	2.9%	87~~.0~~%	58~~.0~~%	84~~.4~~%	49~~8.8~~%
**Provide an estimate on wait time and any updates while waiting**	14	2.7%	78~~.3~~%	44~~3.5~~%	75~~.0~~%	32~~1.8~~%
**Clean & hygienic environment / waiting area**	13	2.5%	87~~.0~~%	45~~4.9~~%	7~~6.~~7%	35~~4.9~~%

**Timely access to sub specialised if needed**	11	2.1%	96~~5.7~~%	61~~0.9~~%	93~~.0~~%	61~~0.5~~%
**Specialised nurses and doctors for children**	11	2.1%	97~~.1~~%	75~~.4~~%	100~~.0~~%	77~~.3~~%
**Review and check children while they are waiting**	8	1.5%	~~8~~90~~.9~~%	62~~.3~~%	93~~.2~~%	61~~.4~~%
**Treating the sicker kids first**	6	1.1%	97~~.1~~%	80~~79.7~~%	100~~.0~~%	79~~.1~~%
**Triage to provide initial care such as pain relief**	5	1.0%	100%	65~~.2~~%	95~~.4~~%	63~~2.8~~%
**Minimal bureaucracy, repetition and administration**	5	1.0%	41~~0.6~~%	20~~.3~~%	~~Xx~~XX	~~Xx~~XX
**Transfer of patients to other areas of hospital to happen quickly, smoothly and without repetition**	5	1.0%	87~~.0~~%	38~~7.7~~%	86~~.0~~%	47~~6.5~~%
**Communicate and update parents when there is a change in staff**	3	0.6%	78~~7.9~~%	41~~.2~~%	72~~.1~~%	44~~.2~~%
**Medical Treatment followed**	3	0.6%	87~~.0~~%	57~~6.5~~%	89~~8.6~~%	61~~.4~~%
**Space for visitors in waiting area and treatment area**	3	0.6%	87~~.0~~%	49~~.3~~%	81~~.4~~%	42~~1.9~~%
**Engage parents/carer in the care of the child**	3	0.6%	94~~.2~~%	61~~0.9~~%	91~~0.9~~%	52~~.3~~%
**Retain experienced and skilled paediatric staff**	2	0.4%	99~~8.6~~%	78~~.3~~%	98~~7.7~~%	68~~7.5~~%
**No medical or nursing students**	1	0.2%				
**Parking spaces that are available and reasonably priced**	1	0.2%				
**Mobile reception in ED**	1	0.2%				
**Ease of access to complete medical records**	1	0.2%				
**Doing everything possible**	1	0.2%				
**Staff that skilled in non-English (Mandarin)**	1	0.2%				
**Evidence based**	1	0.2%				
**Name of staff easily seen**	1	0.2%				
**Outcomes/Safety/Stabilise ***	1	0.2%				
**Dedicated space for Autistic kids**	1	0.2%				
*** One word comments where context can't be determined**						
